# Blockchain Based Decentralized Management of Demand Response Programs in Smart Energy Grids

**DOI:** 10.3390/s18010162

**Published:** 2018-01-09

**Authors:** Claudia Pop, Tudor Cioara, Marcel Antal, Ionut Anghel, Ioan Salomie, Massimo Bertoncini

**Affiliations:** 1 Computer Science Department, Technical University of Cluj-Napoca, Memorandumului 28, Cluj-Napoca 400114, Romania; claudia.pop@cs.utcluj.ro (C.P.); tudor.cioara@cs.utcluj.ro (T.C.); marcel.antal@cs.utcluj.ro (M.A.); ioan.salomie@cs.utcluj.ro (I.S.); 2 R&D Department, Engineering Ingegneria Informatica S.p.A., Via San Martino della Battaglia 56, Rome 00185, Italy; massimo.bertoncini@eng.it

**Keywords:** blockchain technology, smart contracts, demand response, smart energy grid

## Abstract

In this paper, we investigate the use of decentralized blockchain mechanisms for delivering transparent, secure, reliable, and timely energy flexibility, under the form of adaptation of energy demand profiles of Distributed Energy Prosumers, to all the stakeholders involved in the flexibility markets (Distribution System Operators primarily, retailers, aggregators, etc.). In our approach, a blockchain based distributed ledger stores in a tamper proof manner the energy prosumption information collected from Internet of Things smart metering devices, while self-enforcing smart contracts programmatically define the expected energy flexibility at the level of each prosumer, the associated rewards or penalties, and the rules for balancing the energy demand with the energy production at grid level. Consensus based validation will be used for demand response programs validation and to activate the appropriate financial settlement for the flexibility providers. The approach was validated using a prototype implemented in an Ethereum platform using energy consumption and production traces of several buildings from literature data sets. The results show that our blockchain based distributed demand side management can be used for matching energy demand and production at smart grid level, the demand response signal being followed with high accuracy, while the amount of energy flexibility needed for convergence is reduced.

## 1. Introduction

**Figure 1:**
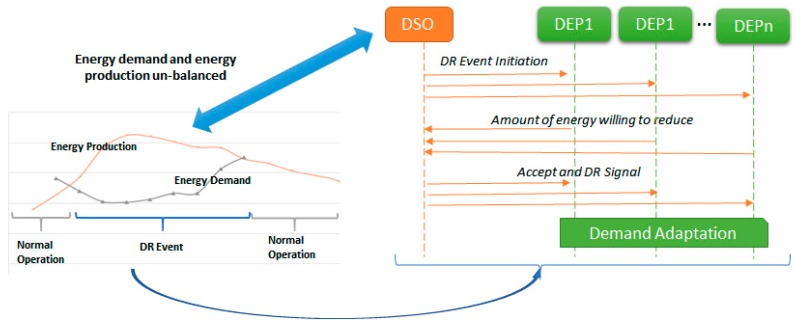
Centralized management of Demand Response (DR) programs in smart grids for energy demand management. DEP = Distributed Energy Prosumers.

Real-time control and supervision play an important role in the smart energy grids management and operation at medium and low voltage levels. Lately, due to the rapid growth in the deployment of Distributed Energy Prosumers (DEPs) the smart grid management problems can no longer be efficiently addressed using centralized approaches, thus, the need for visionary decentralized approaches and architectures is widely recognized [1,2,3,4]. The development of Internet of Things (IoT) smart metering devices together with the prospect of renewable energy integration has increased the level of adoption of decentralized energy networks where, due to the lack of grid-scale energy storage capacity, electrical energy must be used as it is generated [5]. However, the integration of renewable energy has added a level of uncertainty due to the intermittent and unpredictable nature of its generation [6]. Variations in energy production, either surplus or deficit, may threaten the security of energy supply, leading to energy components overload and culminating with power outages or service disruptions. Figure 1 presents such a situation in which, due to an unforeseen peak of renewable energy production in the smart grid, the energy demand and energy production are not balanced. The energy demand is insufficient to cover the entire energy production. The problem is exacerbated by the lack of capabilities for energy storage forcing the Distribution System Operators (DSOs) to frequently curtail (decrease the output of) energy production sources not to endanger the entire grid operation. Of course, this is not an optimal strategy endangering the objectives of increasing the share of renewable energy integration and reducing the emission targets. A better approach to these problems is the demand side management aiming at matching the energy demand with the production by motivating DEPs to shed or shift their energy demand to deal with peak load periods [7,8].

In this context, the DSOs have defined Demand Response (DR) programs providing the possibility for DEPs to play a significant role in the operation of the electricity grid by shaping their energy demand to meet various grid level goals and obtain in exchange financial benefits [9]. Typically, the DSO initiates a DR event at the beginning of a billing period by sending a regulation signal [10,11] ($DR_{Signal}$) to each DEP (see Figure 1) specifying a request to modify the consumption (i.e., a desired energy profile) for a limited period and the associated financial incentives (e.g., bills of credit for participating in the program). The DEPs send bids with the amount of energy they are willing to reduce or to increase their demand while the DSO accepts the bids and checks if the balance between the total energy demand and generation at grid level is met. Afterwards, the DEPs will voluntarily schedule their operation for meeting the agreed profiles by time shifting some tasks that require some amount of electric energy or by switching part of their consumption to alternate sources, such as on-site diesel generators. Thus, DR programs offer several benefits to the energy systems, including increased efficiency of asset utilization and greater penetration of renewables without decreasing stability, by easing capacity issues on distribution networks to facilitate further uptake of distributed generation on congested local networks, reducing the required generator margin and costs of calling on traditional reserve, and including the associated environmental benefits through reduced emissions [12]. 

**Figure 2:**
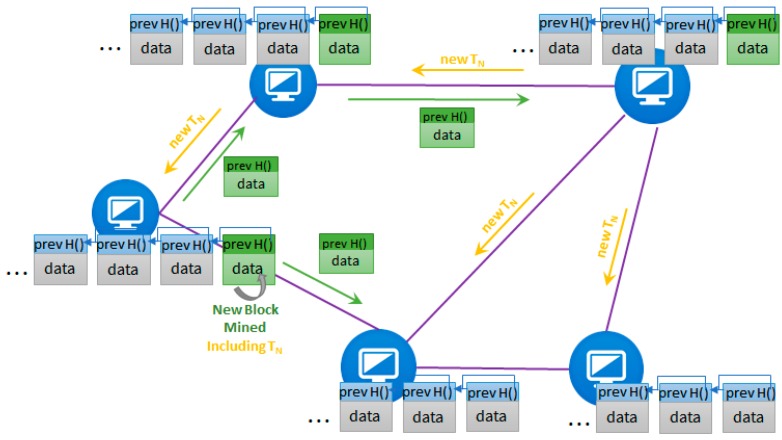
Ledger distribution in peer-to-peer network.

In the last few years, the academic, research, and industrial domains have gained a lot of interest in the distributed ledger and blockchain technology and its potential in decentralizing the management of complex systems. The distributed ledger [13] is composed from a set of blocks, chained back using a linked list of hash pointers, each block storing a set of valid transactions of digital assets (see Figure 2). The linked list is an append-only data structure, thus, any changes that would appear in previous registered blocks would lead to inconsistencies (i.e., the hash pointer of that block would change). If one needs to change the content of a previous block, all the following ones will need to be rehashed and linked to obtain a consistent updated data structure. The advantage brought by this structure is the tamper proof log of all transactional information contained in the blocks. All the transactions and blocks are distributed (i.e., replicated) among the nodes of a peer-to-peer network (see *T_N_* in Figure 2). Registering a new transaction will send it to all its peer nodes and each of them will validate and propagate it further. If any contradictions or invalid states occur, the transaction will not be forwarded. To avoid loops in the network, a node can decide not to forward a transaction if it was already previously registered. Because there is no central authority to create new blocks and each node keeps a local copy of the ledger, consensus algorithms are used to ensure that all the nodes agree upon a global truth about the valid ledger state. The consensus algorithms usually rely on Proof Protocols [14] which define computational intensive problems that are difficult to solve and relatively easy to validate. A new block containing the newest transactions published in the network is mined and validated by a node which finds a solution to that problem (see green block in Figure 2). The block is propagated to all the other nodes in the network to be verified and validated, so any incorrect transactional data or transaction is immediately detected and the block is dropped.

New versions of blockchain technology implementation offer support for the implementation of smart contracts [13]. The smart contracts are pieces of code that implement different business rules that need to be verified and agreed upon by all peer nodes from the network. These contracts are registered in ledger’s blocks and triggered by transaction calls that require each node to update its state based on the results obtained after running the smart contract. Since they are also replicated in all the nodes of the network, they offer a great potential for control decentralization. They act as agents that can have a state and functionality and can be triggered at any point after being successfully deployed, replacing third party middle entities from the transactional world (judges, litigators, escrows, etc.). 

In our vision, the blockchain technology has the potential to provide a disruptive innovative approach to DR programs and energy transactions, paving the way for secured cryptography based decentralized management of smart energy grids. 

In this context, the main contributions of this paper are the following:

- A blockchain based model for distributed management, control, and validation of DR events in low/medium voltage smart grids;
- Blockchain based distributed ledger for storing the data acquired from metering devices as energy transactions in a secured and tamper proof manner;
- Implementation of self-enforcing smart contracts for tracking and checking the compliance of each DEP enrolled in DR programs to the desired demand energy profiles, to calculate associated rewards and penalties, and to detect grid energy unbalances requiring the definition of new DR events;
- Finally, a consensus based DR validation approach to activate the appropriate financial settlement to the flexibility providers and to increase reliability of the smart grid operation.

The main benefits of our blockchain based approach are the distributed management of energy demand, implementation of near real-time automated DR event programs, near real-time financial settlement and events validation, secure energy transactions, and scalability regarding the proportion of distributed generation within the global energy mix. Also, it ensures that energy networks will be more secure and robust because all smart grid nodes will work independently towards the energy grid balancing without centralized supervision and control of the DSO.

The rest of the paper is structured as follows: Section 2 shows related work in the areas of smart grid decentralization and blockchain technology adoption, Section 3 presents the proposed architecture for the decentralized blockchain based energy grid management, and Section 4 presents the smart contracts defined and implemented for DR events management. Section 5 presents the test scenarios and the validation results, while Section 6 concludes the paper and presents future work.

## 2. Related Work

In recent years, the smart grid decentralization has become a topic of research papers aiming to provide an alternative to central entities such as the DSO. Rather than communicating with a single server that collects all data and takes centralized management and makes coordination decisions, a peer-to-peer approach is desired to provide decentralized decision making (i.e., at the level of each DEP), reduced number of messages exchanged with the DSO, and increased network stability. In [15], the authors identify several problems of the current centralized energy management systems. Firstly, due to the centralized approach, which requires a central authority to handle the accounts and the payments, the system’s availability and reliability are jeopardized due to single point of failure threats. Secondly, the same centralized approach makes it possible for a middleman to find patterns of the daily activities of the consumers, leading to low privacy and anonymity in the system. 

In [16], a scalable solution for a fully decentralized microgrid, the Overgrid is presented. The proposed system architecture is a peer-to-peer virtual representation of the physical grid. The nodes communicate using the Gossip protocol, and information about the overall consumption and production profiles is obtained using an average updating scheme. The performance of the network was studied using a simulator of 10,000 nodes with realistic power profiles, and achieved promising results. An experimental validation was conducted using several campus buildings. However, important aspects of decentralization are not discussed, such as Byzantine tolerance, security, and integrity of data. An automated DR program based on Message Oriented Middleware to provide an asynchronous communication paradigm between the network’s components is presented in [17]. The system is not fully decentralized, since the DR programs are considered at the level of energy aggregators and not for each individual DEP part of the smart grid. In [18], the authors propose a multi agent system aiming to provide grid decentralization leveraging on learning techniques. The presented architecture proposes each energy consumption device to be controlled by an intelligent agent which may respond to signals from the network. Each agent learns over time the most suitable set of actions to be taken according to the overall system’s state and following a set of predefined polices (i.e., use available renewable energy, charge battery, etc.). In [19], the authors define a decentralized mechanism for determining the incentive signals in a smart grid using a communication-based decentralized pricing scheme. The proposed mechanism defines and implements a decentralized method to compute the Lagrangian multiplier which is then used for computing the price signal during DR events. However, one of the most notable drawbacks is data privacy. A decentralized price based DR system is presented in [20]. The price signal is internally computed iteratively based on the forecasted energy production and demand ratio and on the user’s willingness to provide load shifting on demand inside an energy sharing zone. The authors of [21] propose algorithms for shifting the individual energy consumption profiles from peak load periods. The centralized approach implements an algorithm for an automation controller that is responsible for inferring the standby consumption of several devices and then computing the maximum monetary reduction. The decentralized algorithm runs in a distributed manner on each smart device, where each device is responsible for its own optimization, without having overview information about the entire system.

Lately, blockchain has been considered as one of the emerging technologies that can be used for developing decentralized grid topologies and enacting the distributed management of energy transactions [22]. The potential benefits of blockchain adoption for smart grids include decentralized peer-to-peer (P2P) energy trading without the need of a third-party intermediary (such as a DSO) and implementation of secured energy transactions and increased share of distributed renewable energy within the energy mix [15]. There are few approaches in the literature that address smart grid decentralized management using blockchain technology. In [15], the PriWatt system is proposed, allowing consumers and producers to trade energy in a peer-to-peer blockchain based network. The energy demand and production are matched through a mediator, in this case, the DSO. The system is assumed to allow a dynamic negotiation of the energy price; however, it is not clear how this is achieved. As in any system based on blockchain, the security and the trust are established through cryptography and consensus algorithms (Proof-of-Work). A similar approach is presented in [23]. NRGCoin is a digital currency defined to implement a decentralized smart contract-based mechanism for rewarding green energy [24]. A producer receives a number of coins proportional to the energy produced, and, similarly, the consumer is billed for the energy consumed. The balance between the energy demand and energy production is held by a DSO substation, which is a node in the network. The price is computed in near real time (every 15 min) by the DSO according to a function dependent on the energy consumption and production rates in the region. A Virtual Distribution Grid to deal with increasing complexity of heterogeneous distribution grid management and related resources is proposed in [25]. The authors focused on the residential sector and used an agent-based hierarchical architecture for demand side, allowing households to transact their surplus/lack of energy with neighbors, aggregators, or DSOs. IoT technologies are used for smart homes interaction while a blockchain based platform is proposed to transact using self-enforcing smart contracts. In [26], the authors emphasize the problems that appear in smart girds due to a central operator and suggest the blockchain technology as a solution to distribute control in every node of the network. They propose a system for managing electricity transactions based on distributed ledgers and smart contracts. Furthermore, they propose to store the monitored data as well in the blockchain. The emergence of IoT leads to de-centralizing systems, thus, traditional business models become deprecated. A new set of business models that could fit the decentralized E-business platform for the IoT is proposed in [27], leveraging on the peer-to-peer blockchain technology. Furthermore, a set of applications of blockchain in the Energy Internet field is presented in [28]. Four scenarios are designed to show its application, such as authentication of carbon emission right, securing cyber-physical systems, trading virtual power sources, and coordinating multi-energy systems.

Several start-ups and projects have started developing solutions in the context of decentralized energy markets based on blockchain. One of them is TransActive Grid [29], which proposes a peer-to-peer application that provides security through cryptography and reliability through consensus. It registers real-time monitored values regarding the generation and usage of energy in a micro grid. Through blockchain technology, the platform enables a secure peer-to-peer energy market and has been demonstrated in a neighborhood of Brooklyn, New York. MIT (Massachusetts Institute of Technology) start-up SolarCoin was implemented based on the DeKo proposal [30], which defined the electricity backed currency in 2011. SolarCoin [31] was developed as a distributed application based on a fork of the Proof-of-Work blockchain framework, Litecoin [32]. It uses 98 billion pre-generated SolarCoins, which are estimated to last for the next 40 years. From 2015, SolarCoin switched to a Proof of Stake (PoS) approach, based on Vericoin [33]. SolarCoin was tested off-grid for a period of 11 months in Tokyo. Similarly, Grid Singularity [34] and Grid+ [35] are developing a ‘pay-as-you-go’ mechanism using blockchain.

In this paper, we will go beyond the current smart grid and demand response management systems by proposing a blockchain based model for distributed control of medium/low voltage smart grids through the setting up and the operation of a decentralized peer-to-peer energy flexibility marketplace, while the control of the energy network remains on the shoulders of a centralized DSO. We propose the use of a grid-level shared and replicated blockchain distributed ledger for storing monitored energy data from IoT smart metering devices and associated energy transactions and self-enforcing smart contracts for DR program’s decentralized management at the level of each DEP and for balancing the grid level energy demand with the actual energy production. Distributed consensus is used for DR verification and financial settlement allowing us to know in near real time the share of energy flexibility activated and shifted.

## 3. Blockchain Based Management of Smart Energy Grids

**Figure 3:**
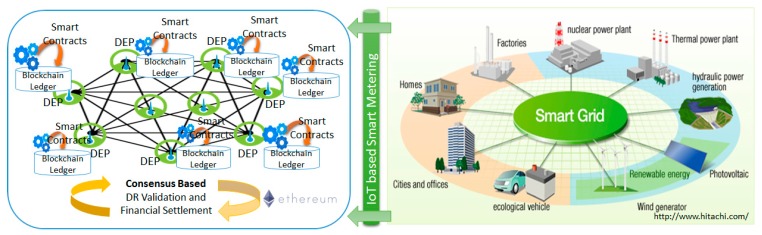
Blockchain based architecture for decentralized management of energy grids.

We propose a blockchain based architecture for distributed management, control, and validation of DR programs in low/medium voltage smart grids (see Figure 3) with a view of assuring high reliability and decentralized operation by implementing trackable and tamper-proof energy flexibility transactions and near real time DR validation. We model the grid as a graph of peer nodes (i.e., DEPs, DSOs, and other interested stakeholders such as Transmission System Operators—TSOs, retailers/suppliers, aggregators) able to coordinate through a blockchain based infrastructure to support fully decentralized energy demand and generation matching to ensure stable grid operation.

A blockchain distributed ledger is constructed and managed at the smart grid level. Each DEP features IoT based energy metering devices and registers the monitored data regarding the energy production or energy consumption values in blocks as part of the ledger. Thus, a DEP is modeled as a node of the peer-to-peer distributed energy network and can maintain a copy of the ledger which is automatically updated when new energy data is registered. 

One of the major obstacles in DEPs engagement in DR programs is data privacy and security, which in this case are innovatively addressed using the blockchain distributed ledger features. The recording of energy transactions in a tamperproof manner is still an open research issue in the centralized approaches, even though a lot of work was put into assuring privacy-preserving smart metering. Efforts are concentrating on providing a trustful bidirectional connection between the DEPs and DSO using protocols such as OpenADR, but system centralization is a key design fault which makes data security a rather sensitive issue [36]. In the decentralized approach, energy data is registered and stored locally in blockchain using DEPs digital identities and then replicated and shared to all the network peers for validation. This is consistent with studies on privacy and security in demand response energy systems that have showed the DEPs preference to keep data in-home and not in centralized data silos [5]. Transaction immutability is another benefit brought by the blockchain technology, ensuring that any energy data registered in the blockchain remains unchanged after its validation. Because the distributed ledger blocks are chained back using a linked list of hash pointers, changing the value of an energy transaction in a block (by an attacker) becomes harder as the number of blocks following that block increases. Considering that in the blockchain based approach all energy transactions are duplicated and shared across the network nodes, it is imperative to provide solid ways of protecting this data. Technically all the information stored as transactions in the distributed ledger is public, thus, to assure privacy preservation, new methods such as zero knowledge proofs are employed allowing one party, named the verifier, to check if the other party, named the prover, has secret information without the prover having to divulge the information [15]. The security-related properties enforced by the distributed ledger technology are the identity, authenticity, and authorization of energy data. Public-key cryptography plays a crucial role in assuring the ownership and security of the transactions. DEPs use their private key to sign their own transactions, which are addressable on the blockchain network via public keys. Being based on mathematical functions that make it easy to compute the public keys, but infeasible to compute the private key given the public key, the cryptographic signature of energy transactions ensure non-repudiation in the blockchain distributed ledger. To enforce ownership, each registered energy data is linked through the transaction to the identity of the DEP, which must own at least one pair of public-private keys. The transfers are guarded with cryptographically locking scripts, the only way to prove ownership, unlock the assets, and perform a transaction is to own the private key pair. At the same time, the private-public keys help build an authentication and authorization mechanism in the distributed ledger. The outputs of energy transactions are not sent directly to an address of the receiver but rather to a script which contains the public key of the recipient. The output script of any transaction contains a set of rules that must be enforced whenever the energy is transacted again in the future. In this way, the assets are locked and the following transaction will need to provide the required input data generated using the private key through an unlocking script. 

A second barrier for demand response management is that a large amount of data must be transported, stored, and processed to effectively control the DR, and this becomes even more critical for real time systems. In the case of centralized management approaches, the integration and interoperability of energy data collected from heterogeneous and distributed DEP is challenging. In the distributed ledger case, the data acquired from the IoT smart metering devices is stored locally in blocks as transactions and replicated for validation to peer nodes. Because the number of transactions can be high, a set of transactions that occur in a short period of time are grouped in a single block and encoded using Merkle Trees featuring hash pointers. This provides increased performance and decreases the length for the chain of blocks and also the size of the blocks to be replicated. All energy transactions in a block are paired two-by-two and the Merkle Tree is incrementally built from bottom to top using the hashes of transactions until the root is reached. The hash of the root encodes the entire collection of transactions that are recorded and aggregated in the block and can be used by the nodes that do not have enough storage capabilities (i.e., light nodes). The light nodes store only the header of the blocks, while the actual transactions are stored remotely. The Merkle Tree root provides enough information for the light nodes to be able to check the consistency of the chain and, given the right input, to check the membership of different transactions in the block. The light node can interrogate other network nodes (i.e., full nodes) for additional information in case it wants to verify if a transaction was mined and to identify the block that stores the actual transaction. At the same time, control and permission mechanisms may be implemented through which the participation of a newly deployed DEP to the distributed network for DR programs management can be accepted or rejected. In this sense, innovative solutions offering a high degree of anonymity could be developed using blockchain platforms such as Quorum (i.e., permissioned implementation of Ethereum supporting data privacy).

**Figure 4:**
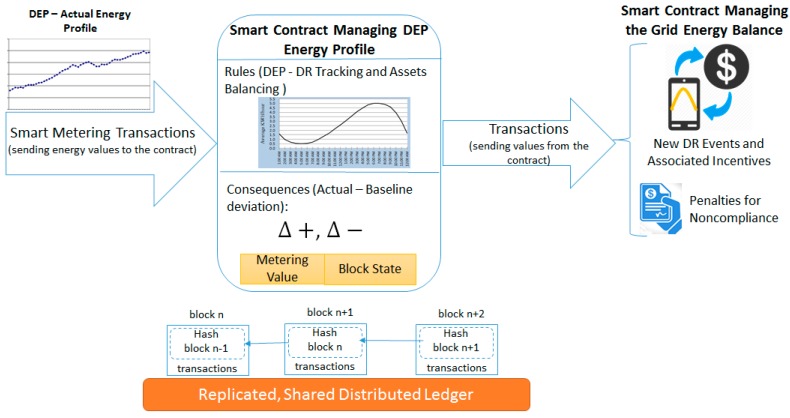
Self-enforcing smart contracts for DR services tracking, energy assets balancing, and decentralized control.

Self-enforcing smart contracts are defined and used to implement in a programmatic manner the levels of energy demand flexibility the DEPs may provide during DR events, associated incentive and penalty rates, as well as rules for balancing the energy demand with the energy production. The self-enforcing smart contracts are defined in a distributed fashion at the level of each DEP voluntarily enrolled with the DR event and specify (see Figure 4) the expected energy demand and adjustments to its energy baseline. For each value provided and stored to a specific block by the IoT smart metering device associated with the DEP, the corresponding smart contract is evaluated by estimating the difference between the expected energy curve and actual monitored energy values (see Section 4.1). If significant deviations are found, actions are taken to rebalance the energy demand with the energy production, thus, smart contracts act as a decentralized control mechanism. Based on the registered deviations in the local grid, new DR events are defined in a decentralized manner, providing also the rates for penalizing the DEP that violates the smart contract and for rewarding the DEP that makes available its energy flexibility (see Section 4.2).

Blockchain distributed consensus is used for DR verification and near real time DR financial settlement using the information gathered from each DEP part of the grid on the share of energy flexibility that was actually delivered. This information is aggregated in blockchain blocks, permanently registered, and replicated across the distributed ledger. Since the data structures offered by blockchain are based on hash pointers, the resulting benefit is that the entire ledger becomes a tamper proof log that can be modified only by re-computing the hashes for all the following blocks, which is infeasible. Thus, an important operation in the blockchain approach is that the network should collectively agree on the contents of the ledger, which in our case reflects the energy state of the grid and the DR events successfully addressed. In our approach, instead of having one authority keeping all energy transactions centralized, like the DSO, the responsibility is equally shared among every peer node of the network. Each time new energy transactions are registered by a DEP, these values are checked by the self-enforcing smart contracts using the DR events agreements and expected energy flexibility levels. Since the smart contracts are deployed in the network, every DR event rule is enforced by each peer and validated in near real time across all the peers. As a result, the decision on the actual share of contracted flexibility, which has been effectively delivered by each peer, and associated financial settlement is unanimously agreed upon by all the other network peers through consensus. They collectively verify the entire blockchain, and energy flexibility transactions are not fully ‘confirmed’ until new blockchain blocks are added. 

To achieve consensus between the nodes, a Proof of Work (PoW) protocol has been used GHOST (Greedy Heaviest-Observed Sub-Tree) together with an ASIC (Application-Specific Integrated Circuit) resistant hashing algorithm (Dagger-Hashimoto) [37]. The PoW is energy consumption intensive, thus, it is not very appealing when we discuss energy efficiency. Therefore, we propose an alternative version based on Proof of Stake (PoS). The PoS consensus cannot be tested yet since the PoS model will be available in Ethereum only at the beginning of next year [38]. In the PoS, the participant stake is used to determine the likelihood of a network peer adding the next block of energy transactions to the blockchain and mining and validating all energy transactions involved in DR and the associated financial settlement. PoS algorithms for mining the next valid block and validating associated transactions/services in the blockchain could be extended to the specific case of DR with a view of providing increased reliability of the DR programs and grid operation. Each DEP part of the grid could take the role of energy transactions validator and could be the miner of the next valid block. Each validator should own some stake in the electricity network, in our case the total rewarded DR incentives received to date, which could be used as a guarantee of the block’s validity. To avoid a centralized decision in which only the richest member makes the validation decision, some degree of randomization should be provided. Solutions like the one provided by PPCoin peer-to-peer cryptocurrency [39], which combines flip coin randomization with the coin age factor (in our case the DR incentives age), could be adopted. Thanks to the blockchain based approach, the valid energy transactions and the actual share of activated energy flexibility (i.e., energy demand deviations from baseline) are known in near real time, and new DR events can be generated to deal with unplanned situations. 

In short, the PoS distributed consensus based DR programs verification works as follows:

(1) The transactions are registered by each DEP and shared with all the other energy parties interested to the same share of flexibility (DSOs, but also TSOs, retailers, etc.) to be validated and mined in future blocks;
(2) the blocks are replicated and the distributed ledger is updated to reflect the state of the grid;
(3) for each DEP, self-enforcing smart contracts check if the share of energy flexibility actually provided matches the expected levels agreed in the DR events;
(4) all DEPs collectively verify the entire blockchain, and the energy transactions are not considered to be fully ‘confirmed’ until they are validated and aggregated in new blocks which are added to the ledger;
(5) the share of contracted flexibility effectively delivered and the financial settlement are calculated using ledger information and the reward/penalty rates defined in the DR events.

The blockchain based management of the smart energy grids provides solutions to many of the problems identified for the traditional, centralized approach as depicted in Table 1. The adoption of the blockchain concepts will transform the smart grid into a democratic community that no longer relies on a central authority, but can take any decision through smart contract rules enforced and verified by each DEP of the grid. Furthermore, the traditional centralized management of the smart grid that is prone to single point of failure vulnerabilities is replaced with a decentralized approach, where the statistics, transactions, control services, and payment settlements are all computed and verified in a distributed manner by each node in the network.

## 4. Smart Contracts for Demand Response Programs

In our approach, a smart contract is a piece of code that defines the expected energy flexibility levels of each DEP for participation in DR programs and the rules for assuring the grid level balance between energy demand and energy production. The rules may describe the behavior of DEPs during demand response events or may even address various constraints for maintaining the grid stability and reliability. These contracts are registered in the blockchain and are triggered by new energy transactions (i.e., registering new energy data from the IoT smart meters), which will make each blockchain node update its state based on the results obtained after running the smart contract. However, even if the term is “contract”, the smart contract should be an agent that has *state variables*, enforces the *associated rules*, and can be triggered at any point after its successful deployment.

### 4.1. Smart Contract Managing the Energy Profile of DEP

The voluntary enrolment of each DEP in a DR event is regulated using self-enforcing smart contracts. Such a contract defines the DEP’s baseline energy profile, current monitored energy values, and expected energy profile, including the expected adjustments in terms of the amount of energy flexibility to be shifted during DR event time intervals (see Table 2). 

The data acquired by each energy metering device associated with a DEP is stored in blockchain and triggers the smart contract execution. We have defined the current energy profile of the DEP as an energy curve to which the current monitored energy values are added:

(1) $$E_{Actual}^{DEP}=\{P_{Monitored}^{DEP}\left(t\right)|\;t\in T\}$$

where $P_{Monitored}^{DEP}\left(t\right)$ is a time series observation [41] of energy demand, *t* is the time index (time at which the value was acquired), and T is the total number of observations. 

As participant in the DR event, the DEP receives from the DSO a control signal ($DR_{Signal}$) through which it is requested to adjust its energy profile to the levels provided by the signal. The $DR_{Signal}$ is calculated by the DSO considering the difference between the total amount of energy production and consumption at grid level, normalized to the baseline energy consumption of each DEP:

(2) $$DR_{Signal}\left(t\right)=P_{Baseline}^{DEP}\left(t\right)+\frac{\left(P_{Demand}^{Grid}\left(t\right)-P_{Production}^{Grid}\left(t\right)\right)}{P_{Baseline}^{DEP}\left(t\right)},\;t\in \left[t_{start},\;t_{end}\right]$$

where $P_{Baseline}^{DEP}$ is the baseline energy demand of a DEP, $P_{Demand}^{Grid}$ and $P_{Production}^{Grid}$ are the total amount of energy consumption and production at grid level forecasted by the DSO, respectively, while $\left[t_{start},\;t_{end}\right]$ represent the interval of the DR event. In Equation (2), the second term of the addition represents the energy demand adjustment to the DEP demand baseline during the DR event.

The provisioning of a DR signal by a DEP is registered in the ledger, thus, the smart contract checks in near real time the monitored energy consumption data against the DR signal to detect any significant deviations and notifies the DSO. The deviations are determined as:

(3) $${∆}_{E_{Actual}^{DEP}-DR_{Signal}}=\sum_{t_{start}}^{t_{end}}\left[P_{Monitored}^{DEP}\left(t\right)-DR_{Signal}\left(t\right)\right]$$

where $\left[t_{start},\;t_{end}\right]$ represents the interval of the DR event. If ∆ value is close to 0, no significant deviation is registered and DEP complies with the DR signal. A positive value (∆+) signals that the DEP has not reduced its energy demand as requested, while a negative value (∆−) signals that the DEP has decreased its energy demand to much.

In case of significant positive or negative deviations (over 10% of the DR signal) the smart contract calculates the associated penalties for DEP. Otherwise the DEP is rewarded considering the DR revenue rates ($R^{Rate}$) and how much of the DEP energy demand profile has been adapted during a DR event. The rewards and penalties are established by the DSO considering the average energy price in the grid. Usually, the penalty rate for under-compliance is the greater than average daily revenue rates (i.e., $P^{Rate}=1.2\times R^{Rate}$) [42]. In our approach, the total penalty for non-compliance is calculated considering the penalty rate ($P^{Rate}$) and the deviation registered for a DEP:

(4) $$DEP^{Penalty}=P^{Rate}\times \left|∆\pm \right|$$

To determine how much a DEP has adapted its energy demand profile (i.e., the degree of this adaptation) to the $DR_{Signal}$, having as reference its Baseline Energy Profile, we used the Adaptability Power Curve (APC) metric defined in the context of the EU (European Union) Smart City Cluster [43]:

(5) $$APC=\frac{\sum_{t_{start}}^{t_{end}}\left|P_{Monitored}^{DEP}\left(t\right)-P_{Baseline}^{DEP}\left(t\right)\right|}{\sum_{t_{start}}^{t_{end}}P_{Baseline}^{DEP}\left(t\right)}$$

where $P_{Baseline}^{DEP}$ is the baseline energy demand of a DEP, and $P_{Monitored}^{DEP}$ is its actual adjusted energy demand during the DR event time interval. APC ranges from 0.0 to 1.0, with 0.0 meaning that the DEP has not adapted its demand to the $DR_{Signal}$ and 1.0 meaning that the energy demand was adapted and its deviation from the $DR_{Signal}$ is zero.

The total incentives for a DEP for its adaptation during a DR event is calculated by the DSO using the formula:

(6) $$DEP^{Incentitives}=APC\times E_{Baseline}^{DEP}\times R^{Rate}$$

where $R^{Rate}$ is the daily revenue rate for each kW of energy shifted established by the DSO or a discount rate on the regular electricity bill.

### 4.2. Smart Contract Managing the Grid Energy Balance

Besides the smart contracts personalized and associated with each individual DEP, we have defined smart contracts that implement the rules for balancing the energy state of the entire grid and enforcing its stability. In other words, it defines the rules for tracking and aggregating the ∆± registered at the level of each DEP with the overall goal of matching and balancing the overall energy production and consumption at grid level. In the case of detecting imbalances between production and consumption, the smart contract initiates new DR events and communicates to the interested DEPs the associated DR Signal and associated incentive and penalty rates. Table 3 presents the state variables controlled by the smart contract.

Equation (7) presents the mathematical formalism for determining the overall Grid Energy State as a balance between the total energy production and consumption calculated by aggregating the ∆± imbalances registered at the level of each DEP of the smart grid:

(7) $$\Pi_{GRID}=\overset{n}{\underset{i=1}{\sum }}{∆}_{E_{Actual}^{DEP^{i}}-DR_{Signal}}$$

where n represents the number of DEPs in the smart grid, and ${∆}_{E_{Actual}^{DEP^{i}}-DR_{Signal}}$ is the deviation from the provided demand response signal registered at the level of ith DEP. 

The Grid Energy State aggregates both positive and negative values. If the result is close to zero ($\Pi_{GRID}\approx 0$), there is a balance between energy production and consumption at grid level, and no further actions need to be taken. Otherwise, a positive value ($\Pi_{GRID}\gg 0$) represents a deficit of energy in the grid (i.e., the total energy demand at grid level is higher than the expected one), while a negative value ($\Pi_{GRID}\ll 0$) represents a surplus of energy in the grid (i.e., the total energy demand at grid level is lower than expected and does not cover the total energy production). If such imbalances are determined, the smart contract constructs new DR signals allowing other DEPs to address them by increasing or decreasing their individual energy demand and, as a result, re-balances the energy state.

## 5. Validation and Results

**Figure 5:**
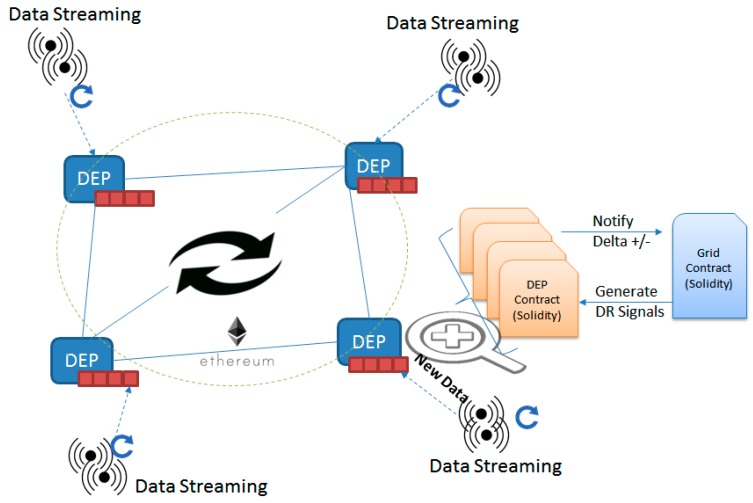
Simulation prototype implementation.

To validate the demand response management decentralization through blockchain and smart contracts, a simulation based prototype was implemented using the Ethereum platform [44] (see Figure 5). The energy consumption data is provided as input of the simulation process considering the energy profiles of different United Kingdom (UK) buildings published by governmental agencies [45]. We considered three types of energy consumers’ profiles, presented in Table 4, to randomly generate different sets of DEPs as peers in our blockchain-based simulation. Similarly, for energy production, we considered two types of renewable profiles (i.e., wind turbines and solar panels). Their characteristics are provided in Table 4, while the weather data (solar radiations and wind speed) are taken from [46] considering the time period and geographic region. The Data Streaming periodically pushes data from the data set as monitored data to the smart contract associated with the corresponding DEP. As a result, all the smart contracts managing the DEPs energy flexibility profiles are updated with the new energy data and checked against the DR signal, and whenever significant ∆± deviations are found, the contract managing the grid’s energy balance is notified.

The smart contracts were implemented using Solidity [51], which is a Turing complete Contract Oriented language. The coin used to pay for energy is the Ethereum coin, Ether, and each client has his own coins, obtained through mining and transactions. 

**Figure 6:**
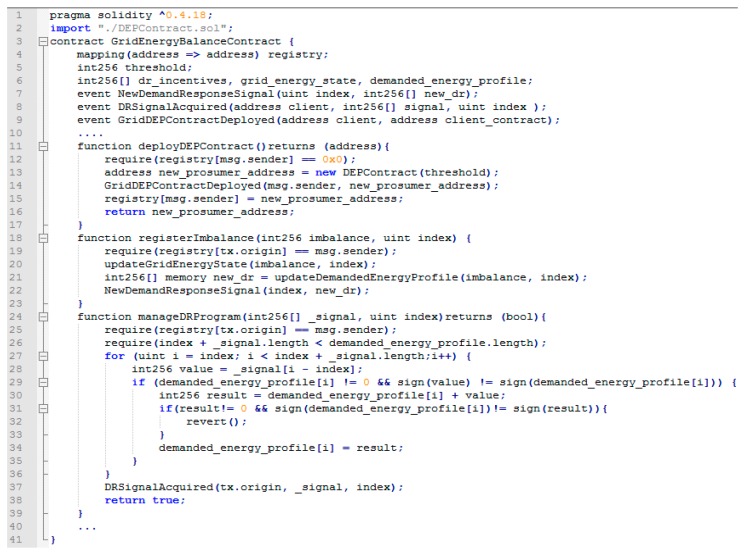
Code excerpt from the smart contract managing the grid energy balance.

The smart contract regulating the grid’s energy balance is deployed once for the entire network and replicated in all nodes (see Figure 6). As a result, all the DEPs in the network register their blockchain in the contract and are able to participate in the validation of the registered energy state of the grid.

The deployment of a new smart contract regulating DEP energy flexibility behavior can be performed only through the functionality exposed by the grid energy balance contract (see line 11 function *deployDEPContract*). This ensures that all the DEPs are kept in the grid’s registry (line 15), so any future actions (registering imbalances, acquiring DR signals) are possible only if the DEPs are already registered by the grid (line 19). Otherwise, any attempt to report imbalances or acquire a DR signal (line 25) are ignored. Whenever an energy imbalance is registered at the level of a registered DEP (∆±), the grid energy state and demanded energy profile variables are updated accordingly (lines 7 and 8) and new DR signals are generated (line 22). As a response to the new DR signal generation, the DEPs may proactively acquire them and participate in the grid energy balancing process. The DR signals that follow are registered through the functionality offered by the smart contracts managing each DEP energy profile, while the grid energy balance contract checks their profiles. If the proposed profiles are not appropriate and generate higher imbalances, the request is automatically reverted (line 32). However, if the profiles are suitable and improve the balance of the grid energy state, the request is registered, and a DR event is issued signaling the promise of the DEP (line 37).

**Figure 7:**
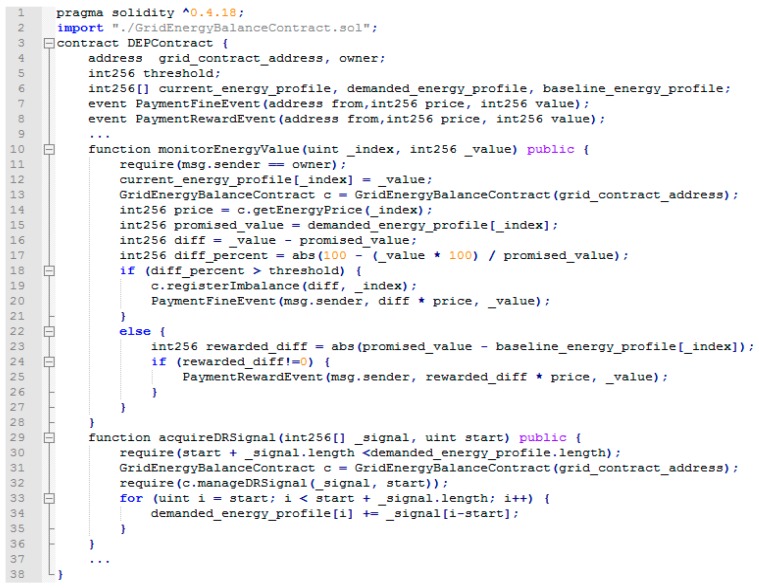
Code excerpt from the smart contract managing DEP energy profile.

Upon the deployment of the smart contracts managing of the energy profile of the DEPs, the contract owner is saved and registered with the grid (see Figure 7), thus preventing the registration of fraudulent monitored values (line 11). The threshold value set during deployment is considered for notifying the grid energy balance contract about the DEPs potential energy deviations from the agreed DR signals. Every 10 min, the latest energy monitored data is registered for each DEP, automatically saved in the current energy profile (line 11), and then checked against the demanded energy flexibility profile (i.e., agreed DR signal). In the case where a difference higher than the threshold is encountered (line 18), the grid automatically is notified about this deviation (line 19). Furthermore, an event is triggered, signaling the obligation of the DEP to pay the fee associated with the imbalance created (line 20). However, if the demanded energy profile was followed (i.e., energy flexibility is actually delivered), and this profile differs from the DEP’s energy baseline, an event is triggered to signal the obligation to reward the DEP based on the amount of energy demand shifted from the original baseline (lines 24 and 25). Also, the contract offers functionality allowing the DEP to express its interests and participate in DR programs by addressing DR signals (see line 29). Upon grid energy contract approval, the DEP updates his energy flexibility profile according to the approved DR signal.

**Figure 8:**
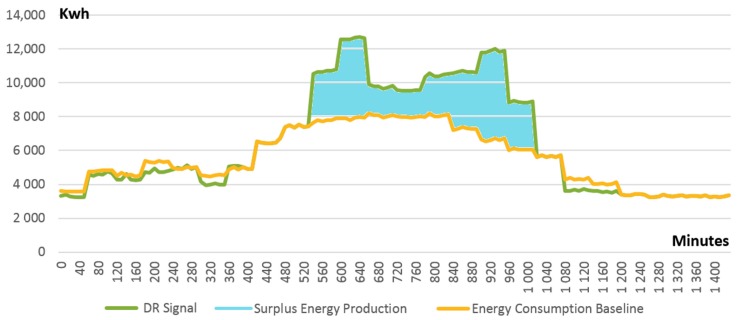
Smart grid energy unbalanced state before blockchain based optimization and planned DR signal.

For evaluation purposes, we simulated a smart grid with 12 DEPs, each of them featuring energy consumption and production profiles randomly selected from UK buildings data sets [45,46] as described above. Figure 8 presents the initial energy state of the simulated grid. It can be noticed that there is a notable imbalance between the energy demand and energy production in the time window of 500 to 1000 min generated by an unpredicted peak of renewable production. Consequently, a DR event is generated by the DSO requesting the interested DEPs to increase their energy consumption demand for a limited period so that a greater share of renewable production is covered. After receiving the request, the DEPs offer their availability for enrolling with the defined DR event. In consequence, adjusted and mutually agreed DR signals (i.e., personalized goal energy demand profiles) are fed into the smart contract managing DEPs individual energy consumption behavior. Each DEP will voluntarily increase its demand by postponing the execution of some tasks that require electric energy for the time interval of the DR event. It is important to note that for successful participation in the DR event, prior to the voluntary enrolment, each DEP needs to evaluate internally and understand how much it can adjust the energy demand. At the same time, how the demand adjustment is obtained is equally important. Will it run some business tasks at peak hours or will it use energy produced on-site? Thus, planning ahead prior to committing to the DR program is mandatory.

**Figure 9:**
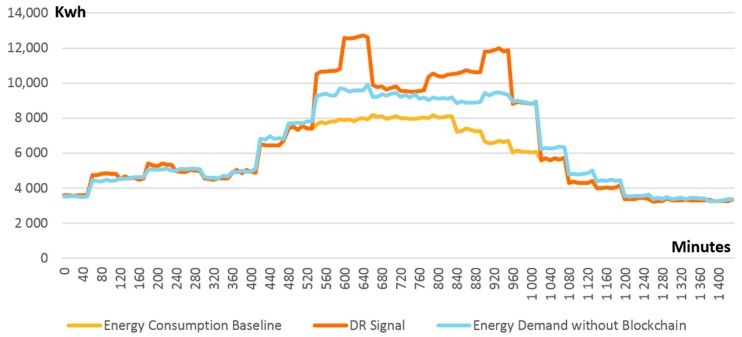
Actual vs. expected energy demand without blockchain based control.

After the start of the DR event, each DEP is monitored by its individual self-enforcing smart contract and if deviations are detected between its energy profile and the agreed DR signal, the smart contract managing grid balance is notified. In our simulation, we considered that not all DEPs involved in the DR program are able to conform to the DR signal and, as a result, deviations are reported. Figure 9 presents the difference between the expected energy demand of all DEPs in the grid (i.e., DR signal in Figure 9) and their actual total energy demand generated by those DEPs not following the DR signals allocated to them (i.e., energy demand without blockchain in Figure 9). Even though a significant amount of energy demand is shifted from the original baseline (i.e., energy consumption baseline in Figure 9) to cover the peak production, since some DEPs do not conform to the agreed DR signal, the production is still higher than consumption. As can be seen in Figure 9, the energy demand has increased by 10% during the DR event but is still under the expected level. 

**Figure 10:**
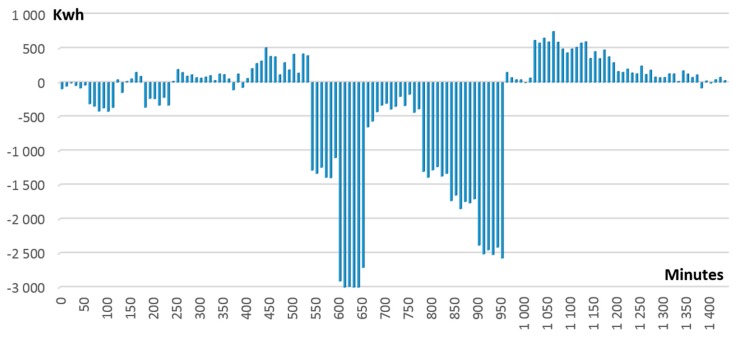
∆± differences reported by smart contracts managing DEPs energy profiles.

In the case of centralized management of demand response programs, these deviations and differences are addressed at the end of the DR event. Using our approach of decentralized blockchain based management and smart contracts, each individual difference (see Figure 10 for ∆± differences in our simulation) is registered by the smart contracts managing individual DEPs energy profiles and reported to the smart contract managing grid energy balance. If ∆ value is close to 0 (e.g., minutes 950 until 1050 in Figure 10), no significant deviation is registered and DEP complies with the DR signal. The positive values (e.g., minutes 1050 until 1200 in Figure 10) mean that the DEP has not reduced its energy demand as requested while negative values (e.g., minutes 550 until 950 in Figure 10) mean that the DEP has decreased its energy demand too much.

**Figure 11:**
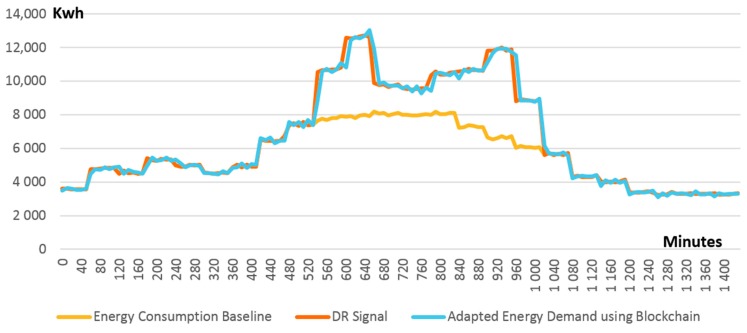
Actual versus expected energy demand using blockchain based control.

The smart contract managing grid energy balance can proactively decide and launch new DR events that could be addressed by other DEPs to correct in a timely manner those ∆± as they are reported. Figure 11 presents the grid energy state as a result of using our blockchain management approach. It can be noticed that our smart contracts-based approach is able to efficiently correct the ∆± by assigning new DR signals to interested DEPs. Consequently, the aggregated energy demand profile of all DEPs is shifted by an additional 7% to match the expected level during the high energy production peak.

Our blockchain based distributed approach focuses on the medium and low voltage grid and is given by electric power distribution design and stress factors which affect the smart energy grid development and management. Traditionally, the high voltage current is converted to medium voltage and is carried out using power lines to the end customers (e.g., medium voltage network setup at city level). Secondary transformers convert the voltage from the medium to a low voltage level, suitable for direct consumption by the end customers (e.g., neighborhood level setup) [52]. From these transformers, low-voltage networks branch off to the customer connections equipped with smart energy metering devices. In addition to this design, several factors stress the energy grid operation: (1) increased share of renewables (solar photovoltaic, wind turbines) which creates more intermittency and volatility in the energy supply, and (2) distributed generation which makes the homes and small scale businesses small energy producers (i.e., DEPs) that are connected to the local low/medium distribution network. This can be problematic at low and medium voltage network levels when local distribution networks and metering systems cannot accommodate reverse flows, or when there are high renewable energy production peaks and not enough energy demand to cover them. For example, in Europe, the total installed capacity of photovoltaic systems reached 69 GW in 2012, 80% of which was connected to low voltage networks [53]. At the same time, Europe remains a worldwide leader with respect to deployed capacity of solar power and with an outlook for growth of over 80% by 2020, which will require novel demand response management solutions as the traditional ones will not be able to scale with the increasing number of DEPs. In certain locations in Italy, 20% of distributed production is fed into the distribution network. In this situation, without a matching demand, the distribution substations struggle to actively manage reverse flows (and ensure overall grid stability). In Germany, solar and wind generation had to be disconnected from the grid at times because renewable energy sources produced a level of power that the grid could not accommodate. In Belgium, the electricity grid had trouble accommodating the production of renewable energy on sunny and windy days when there was not much industrial demand. UK DR and flexibility market is estimated to be only a tenth of its potential size and is struggling to scale fast enough [54]. In this context, our blockchain based approach can provide new solutions ready to drive the change from traditional market approaches and smart grid operations into novel decentralized and community-driven energy systems. At the same time the adoption of blockchain technology for grid management could be the starting point for enabling multi-stakeholder markets as well as market liberalization of district network operation where energy aggregators will combine small scale energy flexibility of DEPs and retailers will be responsible for the market supply of the beyond- electricity commodity to the final customers.

The European demand response management system market is expected to grow from $1.35 billion in 2014 to $6.37 billion in 2019, featuring a growth rate of 36.3% during the forecast period [55]. Market trends for demand response fully correlates with the emerging technology trend, being driven by the growth in intermittent generation, adoption of smart energy metering infrastructures, and highly variable loads which increase the volatility of supply and demand. The increased share of renewables and distribution coincides with the shift towards smart energy metering and electric charging stations and with the pressing need to increase the efficiency of traditional distribution networks. On the demand side, many factors will contribute to increased peak electricity demand such as time, speed, and location of electrical vehicle charging. Satisfying the increase in peak electricity demand caused by vehicle charging will require substantial investment if their charging is unconstrained or uncontrolled. This is particularly the case if neighborhoods with low voltage electricity grids require a high volume of vehicle charging stations, as this will necessitate substantial costly network reinforcements. These developments are leading to a flexible and more decentralized demand/supply load management portfolio, which brings numerous challenges, but in the long term could provide a solution to manage peak demand and grid balancing. The result is not only market price volatility or dramatic swings in net load, but also a reduction in the time to balance the electric system. The time to make critical operating decisions based on rapid changes in the supply and/or demand is decreasing from minute intervals to second intervals. These shorter time scales create significant challenges for effective human interaction in the current decision processes and overall operational system. This includes the short duration responses required to maintain distribution reliability, power quality, and related operational factors, which is not feasible anymore using centralized silos-based DR management approaches. The implementation of smart self-enforcing contracts can bring the necessary level of decentralization in DR events management, allowing for the identification in near real time of significant deviations that may affect grid stability and require demand adaptation. Moreover, the blockchain based technologies have the potential of changing the DR market and management to a fully decentralized one in which individual DEPs will be in charge of controlling the DR programs fulfilment while the financial settlement could be done through consensus and validation of all participants.

## 6. Conclusions

In this paper, we propose a decentralized solution for managing demand response programs in the context of Smart Grids. We integrate the elements of the grid with a blockchain architecture and associated smart contracts to ensure the programmatic definition of expected energy flexibility levels, the validation of DR agreements, and balance between energy demand and energy production. A prototype was implemented in Ethereum to validate and test the blockchain based decentralized management using energy traces of UK building datasets. The results are promising, showing that the grid is capable of timely adjustments of the energy demand in near real time by enacting the expected energy flexibility levels and validating all the DR agreements. Also, it paves the way for setting up a pure peer-to-peer decentralized energy trading mechanism, which will not include any intermediary third party like the DSO, with an impact in term of energy transaction cost reduction. Thus, future improvements will aim to implement multi-stakeholder markets (DSOs, TSOs, retailers as competitors or cooperators for the same energy flexibility) using our blockchain peer-to-peer demand response management platform.

## Figures and Tables

**Table 1:** Blockchain vs. traditional smart grid management.

Issue	Traditional Approach	Blockchain Approach
Single Point of Failure	Yes	No
Energy Profile Anonymity	No	Yes
Payment System	Centralized	Peer-to-peer sales/purchase system
Payment Settlement	By Central Authority	Through Consensus between all nodes
	Up to 60 days [40]	Near real time
Energy Profiles Integration and Aggregation	By Central Authority	Through distributed ledger and consensus between all nodes
Demand Response Programs	By Central Authority	Autonomous signaling through node cooperation and smart contracts
Energy Agreements Verification	By Central Authority	Through consensus between all nodes

**Table 2:** DEP’s smart contract state variables and rules.

State Variable	Description
Baseline Energy Profile ($E_{Baseline}$)	Regular energy profile of a DEP determined as an average of past measured energy values; Reflects how much the DEP would have been consumed in the absence of the DR event.
Current Energy Profile ($E_{Actual}$)	Time series of monitored values acquired by the IoT smart energy metering devices.
Demanded Energy Profile ($DR_{Signal}$)	Signal provided by the DSO through which the DEP is requested to adjust its energy profile to a certain level during the DR event period.

**Table 3:** Smart contract regulating the grid energy balance.

State Variables		Description
Grid Energy State ($\Pi_{\mathrm{GRID}}$)		The balance between energy production and consumption at smart grid level determined as a sum of individual imbalances tracked at the level of each DEP.
New DR Programs	Demanded Energy Profiles for DEPs	New DR signals determined by the DSO for bringing the smart grid into a balanced energy state.
	DR Revenue and Penalty Rates	The rate used to calculate the incentive offered as a reward for following a DR signal. The penalty rate imposed for noncompliance.

**Table 4:** Types of energy consumption profiles considered in our simulation.

Energy Profiles	Node Type	Data Source	
Consumption	Miner	FCO’s (Foreign and Commonwealth Office) King Charles Street Office [47]	
	Regular	Department for Education [48]	
	Regular	National Archives [49]	
Production	Miner	**Solar Panels**	
		Total Solar Panel Area (A)	10,000 m^2^
		Solar Panel Yield (r)	15%
		Radiation Short Wave (H)	From CEH Winfrith [46]
		Performance Ratio (PR)	0.75
		Generated Energy	A × r × H × PR [45]
	Regular	**Wind Turbines**	
		No. Turbines	10
		Blade Length (l)	52
		Wind Wpeed (v)	From Telegraph Hill [46]
		Air Density (q)	From Telegraph Hill [46]
		Power Coefficient (Cp)	0.4
		Generated Energy	0.5 × q × 2Pi × l × v^3^ × Cp [50]
